# Special Issue “Recent Advances in the Food Safety and Quality Management Techniques”

**DOI:** 10.3390/foods13244165

**Published:** 2024-12-23

**Authors:** Anand Babu Perumal, Xiaoli Li, Yong He

**Affiliations:** College of Biosystems Engineering and Food Science, Zhejiang University, Hangzhou 310058, China; yhe@zju.edu.cn

Food quality and safety are essential from the health and fiscal points of view. Nowadays, consumers are demanding safe products with high nutritional value, appealing sensory properties, and a long shelf life [1,2,3]. Keeping environmental, nutritional, and decontamination aspects in mind, many promising technologies have been developed to assess food quality and safety [4,5]. In the food industry, thermal and non-thermal technologies such as the pulsed electric field, high-pressure processing, intense pulse lights, ohmic heating, ultrasound, irradiation, etc., impose less impact on the quality of food and the environment; thus, meeting the requirements of green technology [6,7,8,9]. In recent years, the application of vibrational spectroscopy techniques, such as Raman spectroscopy, hyperspectral imaging, Fourier transform infrared (FTIR) spectroscopy, nuclear magnetic resonance (NMR), etc., has attracted considerable interest in food safety and quality management systems [10,11,12,13,14].

This Special Issue focused on emerging techniques, including thermal and non-thermal, spectroscopic, and imaging techniques; sensors; and other advanced technologies applied to assess the quality and safety of food and agricultural products. In addition, the Special Issue focuses on recent advances in the development of fast and reliable methods for the management of the safety and quality of food and agricultural products.

The following are the six accepted and published papers in this Special Issue:

1. Wang et al. [contribution 1] concluded that the artificial neural network (ANN) model outperforms when compared to SVR and ANN to predict the changes in Matsutake mushroom quality indicators over time in different storage environments. They further enhanced the performance of the ANN model by optimizing it using the Levenberg–Marquardt, Bayesian regularization, and scaled conjugate gradient backpropagation algorithms. The study has significant implications for optimizing existing storage methods to maintain optimal Matsutake mushroom quality and taste during storage and enhance the market competitiveness of Matsutake mushrooms.
2. Chen et al. [contribution 2] tried to determine the regulatory mechanism by which ethylene response factors (ERFs) modulate fruit ripening in bananas. They showed that the overexpression of MaERF012, which is a transcriptional activator, alters the transcriptome profiles of the fruit peel and pulp, and the differentially expressed genes were mainly enriched in starch and sucrose metabolism, plant hormone signal transduction, biosynthesis of secondary metabolism, and fructose and mannose metabolism. The results provide new insights regarding the ERF and transcription factors (TF) family’s role in regulating fruit yellowing and softening.
3. Mollazade et al. [contribution 3] developed a non-contact imaging system using a multispectral imaging-based approach to spatially analyze the quality of pineapple slices. They used filter-based or light-based multispectral imaging systems to create spatial distribution maps of the chemical compositions on the surface of pineapple slices. The prediction of the chemical composition in each pixel of the multispectral images using calibration models resulted in the spatially distributed quantification of the fruit slice, which varied spatially according to the maturation of single fruitlets in the whole pineapple. The calibration models provided reliable responses spatially throughout the surface of fresh-cut pineapple slices with a constant error. This approach to using commercially relevant wavelengths and calibration models could be applied in classifying fruit segments in the mechanized preparation of fresh-cut produce.
4. He et al. [contribution 4] found that the transcription factor StWRKY6 plays a vital role in plant response to environmental stress in potatoes. It regulates the expression of multiple genes, enhancing plant tolerance to toxic metals such as cadmium. This mechanism promotes antioxidant production, enhances photosynthesis and carbon metabolism, and increases NADPH and ATP content in Arabidopsis leaves. This study provides valuable insights into the molecular mechanisms of plant response to environmental stress and offers effective strategies for mitigating heavy metal pollution and ensuring food safety and security.
5. Metzenmacher et al. [contribution 5] developed a contactless ultrasonic wave mode-based method for density measurement in highly aerated batters. The variation in the bubbles in the batter due to alternating mixing times changes the density of the batter. Therefore, by measuring the mode conversion with respect to the acoustic features of the batter, its density can be measured down to >500 g/L. The results yielded a reasonable prediction for highly aerated batters (NRMSE = 6.32%), including for varying batter formulations (NRMSE = 4.15%). This method can be extended and optimized.
6. Yang et al. [contribution 6] developed microbial cultivation and high-throughput sequencing to explore the microbial community dynamics of brown rice during germination. They reported that the germination was significantly influenced by the microbial composition and diversity. They concluded that microbial community dynamics analysis can provide related information to establish control points during brown rice germination and can be useful in ensuring microbial safety in the industry.
